# Comprehensive overview of the toxicities of small-molecule cryoprotectants for carnivorous spermatozoa: foundation for computational cryobiotechnology

**DOI:** 10.3389/ftox.2025.1477822

**Published:** 2025-02-26

**Authors:** Isaac Karimi, Layth Jasim Mohammad, A. Suvitha, Zohreh Haidari, Helgi B. Schiöth

**Affiliations:** ^1^ Research Group of Bioengineering and Biotechnology, Laboratory for Computational Physiology, Department of Biology, Faculty of Science, Razi University, Kermanshah, Iran; ^2^ Department of Medical Microbiology, College of Medicine, Babylon University, Hilla, Babylon Governorate, Iraq; ^3^ Department of Physics, CMR Institute of Technology, Bengaluru, India; ^4^ Department of Surgical Sciences, Division of Functional Pharmacology and Neuroscience, Uppsala University, Uppsala, Sweden

**Keywords:** spermatozoa, cryoprotectant, cryopreservation, seminal plasma, carnivores, thermodynamics, toxicology

## Abstract

**Background:**

The specific and non-specific toxicities of cryoprotective agents (CPAs) for semen or spermatozoa cryopreservation/vitrification (SC/SV) remain challenges to the success of assisted reproductive technologies.

**Objective:**

We searched for and integrated the physicochemical and toxicological characteristics of small-molecule CPAs as well as curated the information of all extenders reported for carnivores to provide a foundation for new research avenues and computational cryobiology.

**Methods:**

The PubMed database was systematically searched for CPAs reported in SC/SV of carnivores from 1964 to 2024. The physicochemical features, ADMET parameters, toxicity classes, optimized structures, biological activities, thermodynamic equilibrium constants, and kinetic parameters were curated and assessed computationally.

**Results:**

Sixty-two relevant papers pertaining to CPAs used in SC/SV were found, and 11 CPAs were selected. Among the properties of CPAs, the molecular weight range (59–758 g/mol), melting point (−60°C to 236°C), XlogP3 (−4.5 to 12.9), topological polar surface area (TPSA; 20–160 Å^2^), Caco2 permeability (−0.62 to 1.55 log(P*app*) in 10^–6^ cm/s), volume of distribution (−1.04 to 0.19 log L/kg), unbound fraction of a CPA in plasma (0.198–0.895), and *Tetrahymena pyriformis* toxicity (log µg/L; −2.230 to 0.285) are reported here. Glutathione, dimethyl formamide, methyl formamide, and dimethyl sulfoxide were used as the P-glycoprotein substrates. Ethylene glycol, dimethyl sulfoxide, dimethyl formamide, methyl formamide, glycerol, and soybean lecithin showed Caco2 permeabilities in this order, whereas fructose, glutathione, glutamine, glucose, and citric acid were not Caco2-permeable. The CPAs were distributed in various compartments and could alter the physiological properties of both seminal plasma and spermatozoa. Low volume distributions of all CPAs except glucose indicate high water solubility or high protein binding because higher amounts of the CPAs remain in the seminal plasma.

**Conclusion:**

ADMET information of the CPAs and extenders in the bipartite compartments of seminal plasma and intracellular spaces of spermatozoa are very important for systematic definition and integration because the nature of the extenders and seminal plasma could alter the physiology of cryopreserved spermatozoa.

## Highlights

• Specific and non-specific toxicities of cryoprotective agents (CPAs) must be considered in assisted reproductive technologies
• Non-specific toxicity is caused by ice crystallization and subsequent thermal cytoinjuries
• Specific toxicity depends on the innate toxic properties of the CPAs and their concentrations
• Small-molecule CPAs are a large category of chemicals used in semen cryopreservation/vitrification (SC/SV)
• The bipartite compartment of seminal plasma and intracellular space of spermatozoa alters the thermodynamic and toxicological features of small-molecule CPAs
• Glycerol and ethylene glycol have been used to prepare semen extenders in carnivorous SC/SV for over 60 years

## 1 Introduction

**FIGURE 1:**
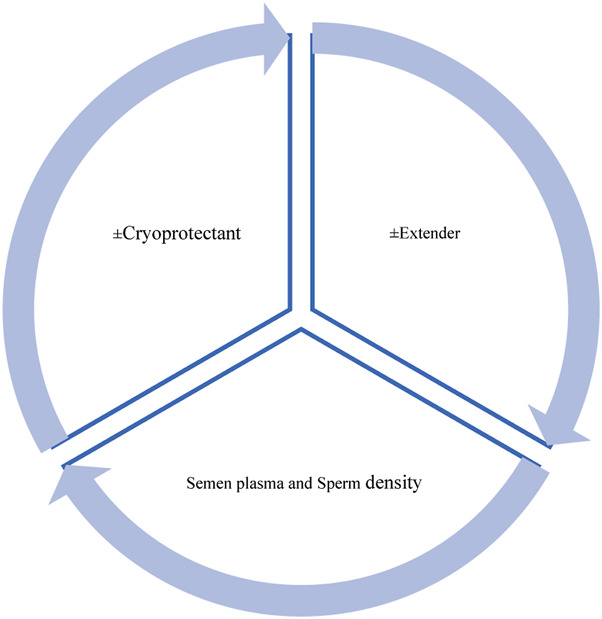
Three modulators of spermatozoa fitness during semen cryopreservation/vitrification.

Semen cryopreservation (SC) and semen vitrification (SV) are two techniques used for long-term biobanking of the male gametes of mammals and other species. These cryobiotechnological methods have been used for more than half a century without considerable progress compared to the methods introduced in the original works (Foote, 1964; Foote and Leonard, 1964), and the original formulations of the semen extenders have often been modified without any systematic testing. Although some process improvements have been attempted on the initial SC recipes, the post-thaw fitness (viability and motility) of spermatozoa do not differ significantly over those of canonical efforts. Therefore, it is important to develop novel interdisciplinary and multidisciplinary methodologies to conserve the viability of spermatozoa during SC/SV or long-term biobanking. Spermatozoa viability is mainly influenced by the semen plasma (SP), extenders, and thermodynamic behaviors of the cryoprotective agents (CPAs; Figure 1). For example, Hyakutake et al. (2015) showed that the motility of bovine spermatozoa differs in the presence of Newtonian and non-Newtonian fluids or extenders; in this context, if the spermatozoa pellets have been separated from SP and rediluted at predetermined concentrations, the exact quality of the extender will strictly impact the fitness of the spermatozoa more than whole semen dilution. Since semen is a non-Newtonian fluid, the mixing (dilution) of whole semen and extenders will produce new properties that the naïve spermatozoa do not have in their prior-experience motion repertoire to swim in this new-content pond. In this regard, Mane et al. (2023) used microchannel methods and showed that the highest motility of spermatozoa is observed in methylcellulose solution that acts as a pseudoplastic non-Newtonian fluid. In summary, understanding the biophysical properties (i.e., microfluidic features) of extenders and their perturbations in the presence of CPAs is critical steps in designing smart extenders or CPAs.

Spermatozoa naturally pass through various survival barriers (intratesticular, urethral, airy, vaginal, uterine, and tubal) with diverse physicochemical and thermodynamic characteristics that may be harsh on their innate motility and viability. Therefore, more biomathematical models and simulations are required to explain the swimming patterns of spermatozoa or spermatozoon communities in various milieus. The outcomes of these investigations could offer new avenues for designing artificial SP as well as a new generation of extenders for precision medicine and personalized treatments of infertility associated with the transport and motility of spermatozoa. Hence, complete understanding of these spermicidal barriers would be the second step in designing smart extenders or CPAs. Finally, the third step in designing smart extenders or CPAs involves dissecting the chemical and physical interactions of all components of semen, spermatozoa, and exogenous additives, and this requires deep understanding of the interactions between the exogenous and endogenous components of semen extenders.

In addition to the aforementioned steps highlighting the smart design of semen extenders, a major bottleneck that must be addressed involves thermal shocks that can lead to cryoinjury (cold-induced injury) and/or pyroinjury (heat-induced injury), which are collectively known as thermoinjuries. In this continuum, the most common thermoinjuries that occur during SC and SV can be summarized as decreasing post-thaw fitness of the spermatozoa, including decreased or altered motility as well as decreased viability. In this context, any alterations such as derangements in plasma membrane integrity (PMI), acrosomal membrane integrity (AcI), mitochondrial membrane potential (MMP) integrity, and DNA integrity as well as morphological abnormalities, percentage remnant cytoplasmic droplets, vulnerability to oxidative or reductive stresses, apoptosis, spermatozoa dyskinesia, zona pellucida disbinding assay, and altered -omes (transcriptome, genome, proteome, metabolome, and physiome) can fulminate to increase male-factor subfertility. To overcome these thermoinjuries, SC and SV optimizations have been pursued, and the application of CPAs is the top priority among all optimization programs to specify the inborn (specific) and acquired (non-specific) toxicities.

Despite the aforementioned requirements to optimize the SC and SV, the study of cryobiotechnological approaches is inadequate. The biophysical and biochemical aspects of semen as a non-Newtonian fluid and its behaviors during freezing as well as the optimum roles of CPAs employed in semen extenders to avoid cellular cryoinjuries during SC and SV (AbdelHafez et al., 2009) must be studied further. In this context, cryoinjury of spermatozoa is a critical and cardinal issue during SC that encompasses derangements of the PMI, AcI, DNA integrity, etc., which decrease the post-thaw fitness of spermatozoa. Luvoni (2006) and Bencharif and Dordas-Perpinya (2020) focused on the non-specific toxicities of CPAs, but evaluation of the specific toxicities of CPAs is an incentive for improving the SC/SV. In such cases, the spermatozoa experience two temperature shocks, namely, cold shock during freezing and heat shock during thawing, both of which can result in ice crystallization or cellular dehydration. To overcome these thermal shocks, we could add the CPAs during freezing and remove them during thawing to decrease the toxicity to the spermatozoa. In summary, despite the introduction of several commercial extenders and CPAs in the market, the specific and non-specific toxicities of CPAs have not been studied in detail.

To the best of our knowledge, there is no available database pertaining to the physicochemical properties of currently available CPAs used for SC/SV of animals that can enlighten researchers on the physicochemical and biological spaces of CPAs and their boundaries (cut-offs) or provide current integrated information on designing/discovering new CPAs. Therefore, the present work focuses on the computational physicochemistry, thermodynamics, and toxicology of small-molecule CPAs employed for the SC/SV of carnivores to aid research on the advantages of computational cryobiotechnology.

## 2 Methods

### 2.1 Systematic review

**FIGURE 2:**
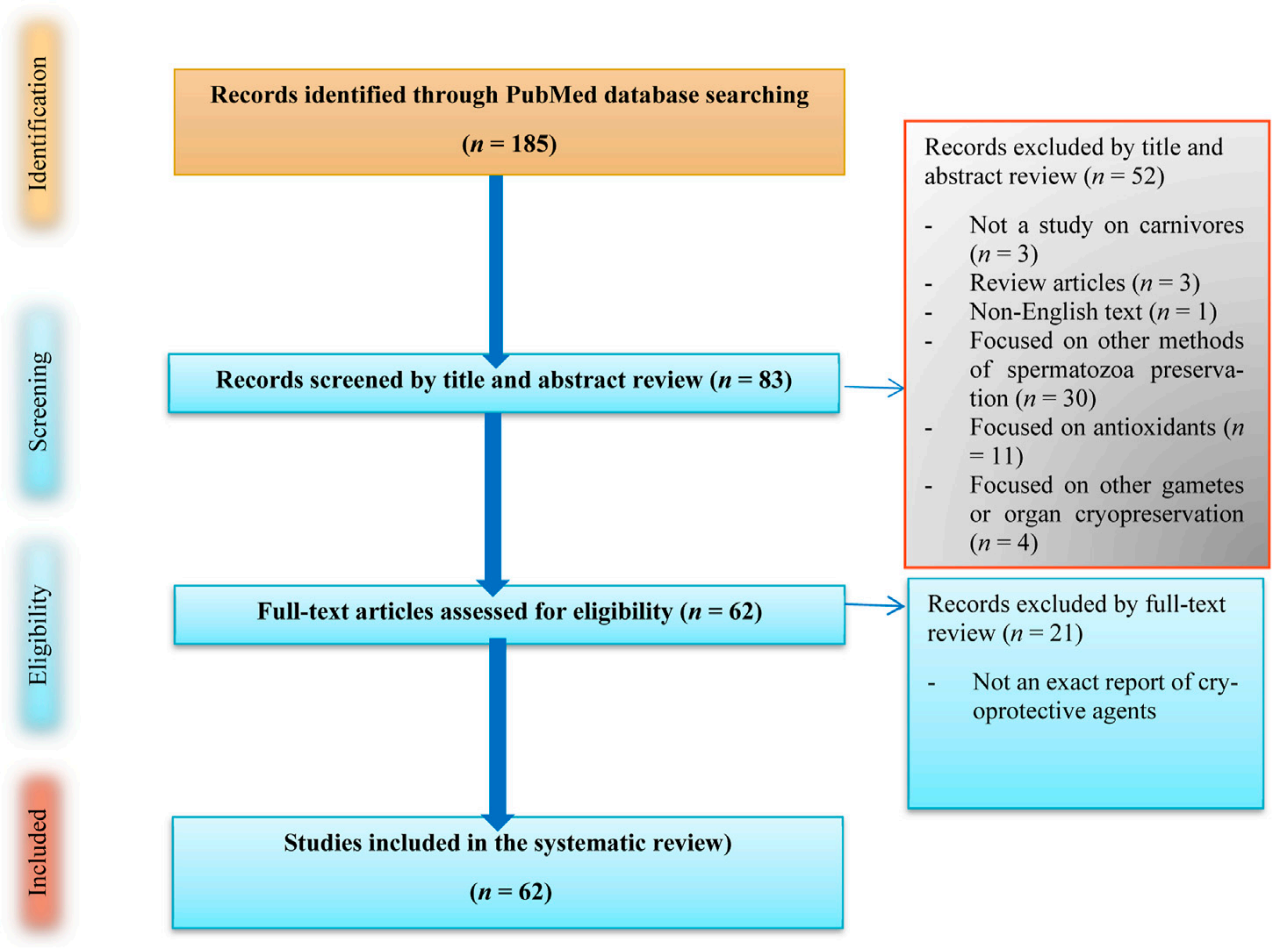
Flow diagram showing the literature search on cryoprotective agents used in carnivorous semen cryopreservation/vitrification.

We comprehensively and historically perused publications regarding carnivorous SC/SV on PubMed dating from 1964 to 10 January 2024 as well as searched for papers using the search string “[(spermatozoa) AND (cryoprotectant)] AND (carnivores)” that adhered to the PRISMA guidelines (Liberati et al., 2009), as demonstrated in Figure 2. These studies were carefully screened, and eligible works were selected based on the following inclusion criteria: (1) studies published in English, (2) studies aimed at reporting carnivorous SC/SV, and (3) studies reporting CPAs and extenders. We excluded papers focused on other animals or the cryopreservation of different organs as well as review articles; however, the references of such works were scrutinized to find relevant works.

### 2.2 Physicochemical and biological features of the selected CPAs

The physicochemical attributes of CPAs used in carnivorous SC were curated from the PubChem database (https://pubchem.ncbi.nlm.nih.gov/). Then, the chemical properties, molecular structures, canonical simplified molecular input line entry system (SMILS), XlogP3, logS, cellular locations, molecular weights in g/mol, melting points in °C, topological polar surface areas (TPSAs; Å^2^), and toxicity classes of common small-molecule CPAs were computed.

A knowledge-based approach based on an additive atom/group model starting from the known logP value of a similar reference compound (Cheng et al., 2007) was employed to compute XlogP3. Here, the logP value is a constant defined as log10 (partition coefficient) with the partition coefficient P = [organic]/[aqueous], where [ ] indicate the concentrations of the solutes in the organic and aqueous partitions. A negative value of logP indicates that the compound has a higher affinity for the aqueous phase (more hydrophilic); when logP = 0, the compound is equally partitioned between the lipid and aqueous phases; a positive value of logP denotes a higher concentration of the lipid phase (i.e., the compound is more lipophilic). For instance, logP = 1 indicates a 10:1 partitioning of the organic:aqueous phases. In contrast to XlogP3, logS is related to the water solubility (mol/L) of a chemical and is expressed as a common solubility unit in log10 value. The molecular polar surface area (PSA) refers to the surface area corresponding to the polar atoms and is a descriptor of the passive molecular transport through the membranes for predicting the transport properties of chemicals (Ertl et al., 2000).

SwissADME (http://www.swissadme.ch/) (Daina et al., 2017) was used to compute the biological activities. The bioavailability radar offers a speedy assessment of the medication resemblance of a particle (Daina et al., 2017). Six physicochemical properties (lipophilicity, size, polarity, solubility, flexibility, and saturation) were computed for each of the CPAs, and these descriptors were established with physicochemical ranges along each axis. The pink areas outline the best possible regions for each of the ranges. Briefly, the fitness parameters include lipophilicity (LIPO) as logP (XLOGP3) values ranging from −0.7 to 5.0; SIZE as molecular weights in the range of 150–500 g/mol; polarity (POLAR) as TPSA values ranging from 20 to 130 Å^2^; insolubility (INSOLU) as solubility with logS (ESOL) values ranging from −6 to 0; insaturation (INSATU) as saturation fractions of carbons with sp3 hybridization (fraction Csp3) ranging from 0.25 to 1; and flexibility (FLEX) as the number of rotatable bonds ranging from 0 to 9 (Daina et al., 2017).

The boiled-egg construction depicts a snapshot of the human intestinal absorption (HIA) and blood–brain barrier (BBB) permeability of a substance (Daina and Zoete, 2016), where the blue dots represent the P-glycoprotein (PGP) substrates (PGP+) and red dots indicate the PGP non-substrates (PGP−). The outer gray area represents compounds with lower gastrointestinal absorption and limited BBB penetration; the white area represents the physicochemical space of the molecule with the highest probability of passive HIA; the yellow area represents the physicochemical space of the molecule with the highest likelihood of BBB penetration.

### 2.3 Thermodynamic equilibrium constant and kinetic parameters

For the most part, a chemical reaction has some basic equilibrium constant parameters; however, these are not effortlessly obtained from experiments. Herein, we show that the computed thermodynamic properties of CPAs used for carnivorous SC are dependent on Chemeo, which includes high-quality compound properties (www.chemeo.com), and the Joback method that produces an anticipated value of 813.3 K. The actuation entropy (S°), enactment enthalpy (ΔfH°), and Gibbs free energy obtained from the thermodynamic properties contrast between the explicit reactant and change states compared to individual response stages subbed into the progress state theory (Benkert et al., 2007), as shown in Equations 1, 2 to gage the rate steady (K) and preexponential factor (A). The thermodynamic equilibrium constant of each response was obtained from the ratio of the forward to retrogressive rate steady values for the resulting correlation.

$$K=k_{B}T/h{\left(p/\text{RT}\right)}^{1-n}\exp\left(S{}^{\circ}/R\right)\exp\left(-\Delta \text{fH}{}^{\circ}/\text{RT}\right)$$ (1)

$$A=k_{B}T/h{\left(p/\text{RT}\right)}^{1-n}\exp\left(n\right)\exp\left(S{}^{\circ}/R\right)$$ (2)

where k_B_ is the Boltzmann constant, h is the Planck constant, and n is the molecularity.

We reported the initial values of all chemical properties of CPAs, from which their proton affinities were computed. The basicity was determined from the presence or absence of the reaction shown in Equation 3 and its inverse reaction, where A is an amino acid, B is a reference base, and AH^+^ and BH^+^ are the protonated versions of the amino acids and bases, respectively (Gorman et al., 1992; PAaJd, 2006).

$$A+{\text{BH}}^{+}\to \;{\text{AH}}^{+}+B$$ (3)

In addition, the optimized structures of the chosen CPA mixtures were predicted using density functional theory (DFT), GaussView (Frisch HBS et al., 2009), and Gaussian 09 program (Frisch et al., 2009) with 6-311++G (d, p) premise sets. The potential limitations of the chosen tools were addressed in the study, and the parameters chosen were essential for the transparency and robustness of the study based on careful consideration of their strengths. For example, SwissADME is known for its ability to predict the absorption, distribution, metabolism, and excretion (ADME) properties, which are crucial for assessing the suitability of a cryoprotectant, and PubChem is a widely used and authoritative resource for chemical information.

### 2.4 ADMET parameters and toxicological features of the selected CPAs

The features of computational toxicology including absorption, distribution, metabolism, excretion, and toxicity (ADMET) were predicted using a web ADMET server (http://biosig.unimelb.edu.au/pkcsm/prediction) (Pires et al., 2015). This allowed determination and expression of the ADMET boundaries through investigation of human influences on medication (pharmacokinetic properties), neutral nature, and the unwavering quality of the restorative media on small-molecule CPAs. Using the SciDaviz package (Benkert et al., 2007), the correlation distribution properties were plotted in graphs in the vertical direction and structured based on expansion with different CPAs.

A set of endpoints including water solubility (log mol/L), colorectal adenocarcinoma cells (Caco2) permeability (log(Papp) in 10^–6^ cm/s), HIA (% absorbed), skin permeability (logKp), PGP substrate, PGP I inhibitor, and PGP II inhibitor was computed to predict the various absorption components of the ADMET assay (https://biosig.lab.uq.edu.au/pkcsm/theory). For instance, using the *in vitro* absorption data of 674 compounds, pkCSM predicts the apparent cellular permeability coefficient of the compound of interest. For the pkCSM predictive model, high Caco2 permeability translates to values greater than 0.9.

Another set of endpoints including steady-state volume distribution (VDss; human; log L/kg), unbound fraction (human; Fu), BBB permeability (log BB), central nervous system (CNS) permeability (logPs), and total clearance (log mL/min/kg) was computed to predict the various distribution and excretion components of the ADMET assay (https://biosig.lab.uq.edu.au/pkcsm/theory). A set of substrates and inhibitors of the cytochrome (CYP) class including the CYP2D6 and CYP3A4 substrates as well as inhibitors of CYP1A2, CYP2C19, CYP2C9, CYP2D6, and CYP3A4 were computationally assessed to predict the metabolism part of the ADMET assay (https://biosig.lab.uq.edu.au/pkcsm/theory). An array of toxicological endpoints, including AMES toxicity maximum tolerated dose (MTD; human; log mg/kg/day), Ether-à-go-go-related gene (hERG) I inhibitor, hERG II inhibitor, rat oral acute toxicity (LD50 mol/kg), rat oral chronic toxicity (LOAEL; (log mg/kg-bodyweight/day), hepatotoxicity, skin sensitization, *Tetrahymena pyriformis* toxicity (log µg/L), minnow toxicity (log mM), and toxicity class, were then computed to predict the various toxicity components of the ADMET assay (https://biosig.lab.uq.edu.au/pkcsm/theory).

Furthermore, a set of toxicological endpoints was employed to show the ecofriendliness of the CPA of interest. For instance, the AMES test is a globally accepted bacterial assay for evaluating the potential genotoxicity, mutagenicity, and even carcinogenicity of chemicals (Zeiger, 2019). The relevancy of the AMES test for SC/SV requires further investigation; however, CPA remnants can alter the reproductive microbiota of packs or pets that have received AI, especially if repeated services have been attempted. This relevancy can be examined by measuring the ability of the CPA to induce reverse mutations at selected loci in several bacterial strains. The cardiotoxic hERG binding of a CPA may seem irrelevant to its evaluation. The human hERG codes for the alpha subunit of the potassium ion channel known as K_v_11.1, which is dubbed so because of its role in coordinating the electrical activity of the heart. The flexible nature of hERG to bind to various ligands was used in its selection as a target in the ADMET assay of all compounds to avoid cardiotoxicity (Garrido et al., 2020). Skin sensitization is a predictor of potential adverse effects such as dermatitis for chemicals applied to the skin. The MTD is commonly estimated as the highest dose that can be administered for a specific duration that will not compromise the survival of the animal through causes other than carcinogenicity (Shayne, 2024). Itis noted that MTD is sometimes also referred to as the minimum toxic dose. The acute median lethal dose (LD50) is the dose of a chemical that will kill 50% of the test organisms within 24 h of exposure. Acute toxicity studies are usually conducted for various routes of administration in rodents to provide the baseline values for novel chemicals of interest (Gadaleta et al., 2019). The computation model for LD50 was built on over 10,000 compounds tested in rats and predicted in units of mol/L. In addition, chronic studies such as rat oral chronic toxicity aim to identify the minimum dose of a substance that results in the least observable adverse effect level (LOAEL) and maximum dose for the no observable adverse effect level (NOAEL). This predictor was built using the LOAEL library comprising 567 compounds.

*Tetrahymena pyriformis* toxicity (log µg/L) is a globally accepted screening test for the detection of environmental toxicants (Maurya and Pandey, 2020). Here, the laboratory-adopted protozoan *Tetrahymena pyriformis* is the most commonly used ciliated model for primary toxicological research, and the dose that inhibits 50% of its growth is the toxic endpoint, which has been identified as values > −0.5 log µg/L. At a higher organism level, minnow toxicity is used to predict the lethal concentration (LC50) of any chemical that would kill 50% of flathead minnows; here, LC50 values below 0.5 mM (log LC50 <-0.3) indicate high acute toxicity. Finally, based on the hepatic injuries induced by 531 compounds, pkCSM computationally predicts the hepatotoxicity of the compound of interest.

The Toxtree v.3.1.0-1851-1525442531402 software platform (http://toxtree.sourceforge.net/) was employed to predict the toxicity class based on the Cramer rule (Cramer et al., 1976; Munro et al., 1996; Patlewicz et al., 2008). Here, the toxicological hazard of oral administration is estimated from the molecular structure, and three classes are defined as follows. Low oral toxicity or Class I substances have simple chemical structures and efficient modes of metabolism; here, the no observable effect level (NOEL) at the fifth percentile is 3.0 mg/kg-bodyweight/day and human exposure threshold is 1.8 mg/person/day. The NOEL is the highest dose or exposure level of a chemical that produces no noticeable (observable) toxic effects. Intermediate oral toxicity or Class II substances possess structures that are less innocuous than Class I substances but do not contain structural features suggestive of toxicity; here, the NOEL at the fifth percentile is 0.91 mg/kg-bodyweight/day and human exposure threshold is 0.54 mg/person/day. High oral toxicity or Class III substances have chemical structures that permit no strong initial presumption of safety or may even show significant toxicity or have reactive functional groups; here, the NOEL at the fifth percentile is 0.15 mg/kg-bodyweight/day and human exposure threshold is 0.09 mg/person/day. The hazardous substances data bank (HSDB) at https://www.nlm.nih.gov/toxnet/index.html is a reliable index for deciphering the biosafety of extenders and CPAs used in the SC/SV of carnivores. The toxin and toxin target database (T3DB; http://www.t3db.ca/toxins/) was employed to compute the other toxicological features.

### 2.5 Statistical analysis

The data were described in terms of mean ± standard error of the mean (SEM) for all features of the selected CPAs in this study. Furthermore, all descriptive statistics of the numeric features were analyzed using IBM SPSS Statistics ver. 20 (https://www.ibm.com/spss).

## 3 Results

### 3.1 Systematic review of carnivorous CPAs

First, 185 articles were screened, and no duplicate works were found. Subsequently, the publications were vetted by title revision, and three review articles were excluded. Thus, 182 articles were selected based on the titles, including those concerned with *in vivo* experiments, research linked to female animals, and toxic substances, along with publications involving species such as humans, mice, and bulls (Figure 2). Among the remaining articles, 83 full-text articles were assessed and discussed by the coauthors to find the best and most effective CPA candidates for dogs (Table 1). Finally, 62 full-text appropriate papers on SC/SV as well as their physicochemical and some toxicological properties of small molecules were discussed (Table 1).

**TABLE 1:** Categorization of studies dedicated to the addition of cryoprotectants to the spermatozoa or semen cryopreservation/vitrification of carnivores.

Study	Carnivore species	Extender and cryoprotectant
Peña and Linde-Forsberg (2000)	Dog	Equex STM paste (Gly; 3, 5, 7%)/TRIS-glucose-egg yolk (EY)
Holst et al. (2000)	Dog	Equex STM paste-/TRIS-citric acid–glucose extender containing 20% EY and 3% (v/v) Gly (EYT-G)
Songsasen et al. (2002)	Dog	Gly, ethylene glycol (EG), or dimethyl sulfoxide (DMSO); glucose, galactose, or fructose/EY-citrate extender; Kenney skim milk extender; glucose and bovine serum albumin (BSA)
Crosier et al. (2006)	Cheetah	Gly/test yolk buffer (TYB)
Nöthling et al. (2007)	Dog	Homologous prostatic fluid/Biladyl^®^ with Equex STM paste^®^ (BilEq) extender and other Andromed^®^
Hermansson and Axnér (2007)	Cat	EY (EYT)/TRIS buffer (TRIS)
Martins-Bessa et al. (2009)	Dog	Taurine (T) and hypotaurine (H) with different levels of calcium ionophore/Uppsala Equex extender (UE)
Chatdarong et al. (2009)	Cat	EY/TRIS extender
Bencharif et al. (2010)	Dog	Low-density lipoprotein (LDL)/TRIS EY and Equex STAMP^®^
Futino et al. (2010)	Dog	Gly, methyl formamide (MF) and dimethyl formamide (DMF)/EY-TRIS extender
Zambelli et al. (2010)	Cat	Equex STM Paste^®^/TRIS-glucose-citrate EYE
Mota Filho et al. (2011)	Dog	Dimethyl formamide (DMF)/ACP-106C^®^ + Gly/10% EY
Sánchez et al. (2011)	Dog	Sucrose plus human tubal fluid (HTF) and BSA
Bencharif et al. (2013)	Dog	LDL and glutamine/EYE
Villaverde et al. (2013)	Cat	Gly/EYE
Alvarez-Rodríguez et al. (2013)	Bear	Heat shock protein A8 (HSPA8), Gly/N-[tris(hydroxymethyl)methyl]-2-aminoethanesulfonic acid-TRIS-Fru with 20% EY
Vizuete et al. (2014)	Cat	Sucrose-based extenders
Johnson et al. (2014)	Wolf	DMSO, sucrose, Gly/Ham’s F10 medium
Yu (2014)	Dog	Glucose/TRIS
Setyawan et al. (2015)	Dog	Gly or EG/TRIS [hydroxymethyl] amino methane, citric acid, glucose, Na–benzyl penicillin, and streptomycin sulfate in distilled water, 40% (v/v) EY, and 10% (v/v) CPAs
Buranaamnuay (2015)	Cat	EY-TRIS medium (EYT) + Gly/Equex^®^
Kurniani Karja et al. (2016)	Tiger	Glucose, lactose, and trehalose/EYE
Belala et al. (2016)	Dog	LDL, liposomes/EY plasma (EYP)
Lucio et al. (2016)	Dog	Reduced glutathione (GSH), Gly/TRIS-yolk-citrate extender
Corcini et al. (2016)	Dog	EYP/EY
Axner and Lagerson (2016)	Dog	Soybean lecithin (Sln)/TRIS-based extenders
Sánchez-Calabuig et al. (2017)	Dog	EY and Sln
Rahman et al. (2017)	Dog	Glucose, Fru/Gly-free TRIS (GFT)
Khan et al. (2017)	Dog	Cholesterol-loaded cyclodextrin, Gly/EY
Brusentsev et al. (2018)	Cat	CaniPlus Freeze (CPF) and SpermFreeze (SF)/EY
Franklin et al. (2018)	Wolf	Sugar source (glucose, Fru, or combination) CPA (Gly or DMSO)/TRIS-glucose-Gly, EY
Van den Berghe et al. (2018)	Dog	Equex STM
Dalmazzo et al. (2018)	Dog	Sln/EY-TRIS
Inanc et al. (2018)	Dog	Cholesterol-loaded cyclodextrin/TRIS
Rahman et al. (2018)	Dog	Glucose-Fru or sucrose/GFT
Caturla-Sánchez et al. (2018)	Dog	Sucrose and/or trehalose/TRIS, citric acid, and glucose: EY (TCG-20% EY) extender
Dalmazzo et al. (2019)	Dog	Sln/EY
Nguyen et al., 2019 Nguyen et al., 2019	Dog	Sln/EY
Belala et al. (2019)	Dog	LDL/liquid or lyophilized EYP
Kawasaki et al. (2020)	Dog	Quercetin/skim milk
Abe et al. (2020)	Dog	Skim milk (SM)/EY
de Sousa Barbosa et al. (2020)	Cat	Gly/powdered coconut water (ACP-117c) or TRIS
Sun et al. (2020)	Dog	Butylated hydroxytoluene (BHT)/TRIS-based extender
Hernández-Avilés et al. (2020)	Dog	DMF or Gly/TRIS-Fru-citrate-EY-based extender
Madrigal-Valverde et al. (2020)	Cat	Gly, dimethylacetamide (DMA), and DMF/TRIS-EY
Gloria et al. (2020)	Dog	EY
Wang et al. (2020)	Panda	Gly/skim-milk-based (INRA96) versus EY-based (TEST) extenders
Silva et al. (2020)	Jaguar	Gly/TRIS, powdered coconut water (ACP-117c), EYEs
Erdmann et al. (2020)	Margay	Gly/EY-based (test yolk buffer; TYB) and EY-free extender (AndroMed; MED)
Hamad Talha et al. (2021)	Dog	Amino acids (essential and non-essential)/EY-free polyvinyl alcohol (EY-free PVA) extender
Galarza et al. (2021)	Dog	Tris, citric acid, glucose +20% EY (TCG-EY) extender
Cheema et al. (2021)	Dog	Penetrating intracellular CPAs, i.e., GLY, EG, propylene glycol (PG), DMF, and methylacetamide (MA), and extracellular CPAs, including, EY, EYP, LDL, and coconut water (CW) in Tris-citric acid-Fru buffer (T) for SC
Schäfer-Somi et al. (2021)	Dog	EYP instead of EY in a TRIS-based Equex STM Paste freezing extender system plus/minus lecithin and catalase
Lima et al. (2021)	Cat	EG 40% or GLY 40%
Hermansson et al. (2021)	Dog	Tris-based extender, containing either 20% EY, 1% Sln type II-S or 1% Sln type IV-S
Tebet et al. (2022)	Cat Ocelot	(TRIS/glucose/EY and commercial BotuCRIO^®^) extender
Strzeżek and Reksa (2022)	Dog	EY from different avian species (hen, goose, and quail)
Talha et al. (2022)	Dog	SyntheChol is a new synthetic, non-animal-derived cholesterol
da Silva et al. (2022)	Dog	Nanoparticles from EYP
Divar et al. (2023)	Dog	EY lipoprotein granules
Cavalcanti et al. (2023)	Dog	INRA-96^®^ extender
Alcaráz et al. (2023)	Cat	Antifreeze protein type I (AFP I) in Tris buffer extender

Note: Glycerol (Gly); ethylene glycol (EG); egg yolk (EY); Gly-free TRIS (GFT), EY extender (EYE); soybean lecithin (Sln); TRIS-glucose-citric acid (TGC); low-density lipoprotein (LDL); sperm cryopreservation (SC).

In summary, Table 1 shows an array of extenders employed in the SC/SV of carnivores. Although various extenders have been commercially distributed as chemically defined media with unknown or lesser-known chemical compositions (e.g., INRA-96 extender^®^), many of the commercial blends contain materials ranging from amino acids to biological fluids such as skim milk. SC is an essential biotechnology in canine reproduction, and several studies have attempted to introduce novel CPAs and/or cryopreservation methods to achieve better results, which have been systematically reviewed herein (Table 1). Readers are referred to the references mentioned in Table 1 for detailed comparisons of the SC and SV methods along with the CPAs and their major roles in decreasing cryoinjuries.

### 3.2 Physicochemical and biological features of the selected CPAs

The cellular locations of small-molecule CPAs are predicted and presented in Table 2. Briefly, none of the CPAs were localized in the cell membrane, while ethylene glycol (EG), citric acid (CA), glutathione (GSH), dimethyl formamide (DMF), and dimethyl sulfoxide (DMSO) were localized in the cytoplasm. Moreover, EG, glycerol (Gly), fructose (Fru), glucose (Glc), CA, glutamine (Gln), GSH, DMF, methyl formamide (MF), and DMSO were localized in the extracellular compartments. Some CPAs were also localized in the mitochondria (e.g., Gly, CA, Gln, and GSH), lysosomes, Golgi apparatus (e.g., Glc), and endoplasmic reticulum (e.g., GSH and Glc). All physiochemical properties of the CPAs may influence their transfer through membranes. The physicochemical characteristics of some selected small-molecule CPAs used in carnivorous SC/SV were curated from databases to obtain cues regarding their behaviors and toxicities.

**TABLE 2:** Physicochemical characteristics of selected small-molecule cryoprotectants used in carnivorous semen cryopreservation/vitrification based on the PubChem database (https://pubchem.ncbi.nlm.nih.gov/) and T3DB (http://www.t3db.ca/toxins/).

Name	Chemical properties	XlogP3	LogS	Location*	Molecular weight (g/mol)	Melting point °C	TPSA Å^2^
Ethylene glycol	Polyol antifreeze and de-icing alcohol (ethanediol); P*	−1.4	0.7	C, EC	62.07	−12.7	40.5
Glycerol	Polyol: Organic polyol plasticizing agent; P*	−1.8	0.83	EC, MI	92.09	18.2	60.7
Soybean lecithin	Botanical: air sensitive. Incompatible with strong oxidizing agents; NP*	12.9	−15.5	—	758.1	236	111
Fructose	Sugar: free monosaccharide; NP*	−2.8	0.79	EC	180.16	103	110
Glucose	Sugar: monosaccharide; P*	−2.6	0.64	EPR, EC, GA, L	180.16	146	110
Citric acid	Excipient, antioxidant, acidulant, and anticoagulant; P*	−1.7	−0.54	C, EC, MI	192.12	153	132
Glutamine	Inhibitor, metabolite, and micronutrient; P*	−3.1	−0.55	EC, MI	146.14	185	106
Glutathione	Antioxidant, antiaging agent; P*	−4.5	0.97	C, EPR, EC, MI	307.33	195	160
DMF	Polar aprotic solvent and hepatotoxic agent; P*	−1	1.07	C, EC	73.09	−60.4	20.3
Methyl formamide	Polar aprotic solvent and hepatotoxic agent; P*	−1	0.84	EC	59.07	−40.0	29.1
Dimethyl sulfoxide	Non-narcotic analgesic, alkylating agent, radical scavenger, and polar aprotic solvent; P*	−0.6	−0.08	C, EC	78.14	18.5	36.3
Dimethylacetamide	Antitumor agent, clear colorless liquid with ammonia-like faint odor and same density as water; P*	−0.8	1.31	C, EC	87.12	20.0	20.3
Methylacetamide	Less-toxic cryoprotectant; P*	−1.1	1.16	EC	73.09	28.0	29.1

Note: EC: extracellular; GA: Golgi apparatus; C: cytoplasm; EPR: endoplasmic reticulum; L: lysosome; MI: mitochondria; XlogP3: predicts the logP value (octanol: water partition coefficients) of a query compound using the identified logP value of a reference compound; LogS: predicts intrinsic water solubility of uncharged compound in water between 20°C and 30°C; P*: penetrating; NP*: non-penetrating cryoprotectant; TPSA: topological polar surface area.

The TPSAs of both EG and Gly were less than 100 Å^2^ (Table 2); therefore, they can cross the cell membrane more easily than CPAs that have TPSAs larger than 100 Å^2^. The TPSAs of the CPAs used for carnivores ranged from 20.3 (DMF) to 160 Å^2^ (GSH), and we cannot categorize the CPAs based on their TPSAs because they do not represent a rule of thumb (Table 2). The hydroxyl groups of EG or Gly make them polar substances that penetrate the cell membrane because of their low molecular weights. Two novel acetamide derivatives (methylacetamide and dimethylacetamide) are also used as CPAs for carnivorous SC/SV (Table 2).

The physicochemical attributes of CPAs used for carnivorous SC/SV are presented in Table 2, and the descriptive statistics of their quantitative features are shown in Supplementary Table S1. Based on the XlogP descriptor, all CPAs except soybean lecithin (Sln) show negative values, meaning that they are hydrophilic; however, they are considered permeating CPAs. The degree of hydrophilicity ranges from −4.5 for GSH to −0.6 for DMSO (Table 2). Among the CPAs, Sln showed an XlogP value equal to 12.9, which reflects the supralipophilic nature of this non-permeating CPA.

Based on the PubChem information, the CPAs were distributed in various cellular locations. All CPAs are distributable in the extracellular compartment as their logS values support this finding (Table 2). The organelle distribution of CPAs can alter all functional aspects of spermatozoa during the thawing and/or freezing phases of SC/SV (Table 2). The logS values of small-molecule CPAs ranged from −15.5 for Sln as the most hydrophobic to 1.33 for DMA as the most hydrophilic agent (Table 2). The molecular weights of the CPAs vary from 59.07 for MF to 758.1 for Sln, while the melting points range from −60.4°C for DMF to 236°C for Sln (Table 2).

**FIGURE 3:**
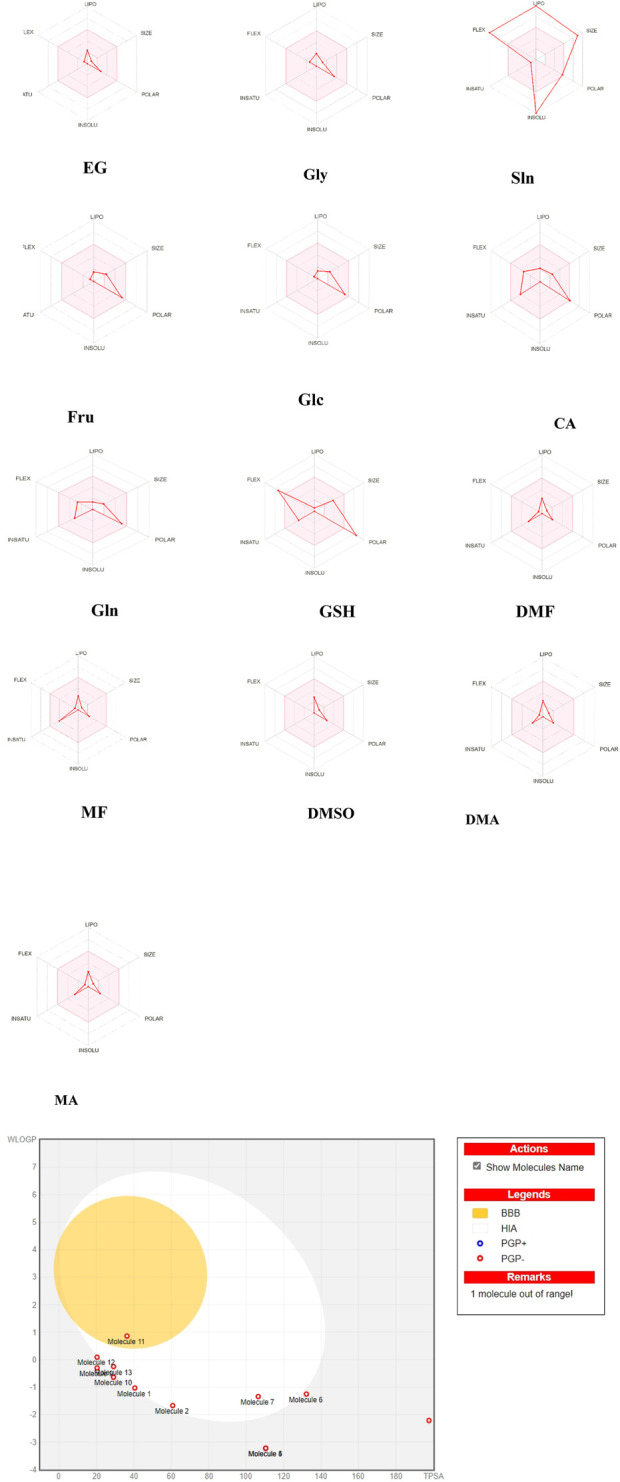
Bioavailability radar of the cryoprotectants used in carnivorous semen cryopreservation based on SwissADME (http://www.swissadme.ch/). The pink areas represent the optimal ranges for the physicochemical properties (size: molecular weight between 150 and 500 g/mol, lipophilicity: XlogP3 between −0.7 and +5.0, polarity: topological polar surface area (TPSA) between 20 and 130 Å^2^, saturation: fraction of carbons in the sp^3^ hybridization not less than 0.25, solubility: logS not higher than 6, and flexibility: no more than nine rotatable bonds). Human intestinal absorption (HIA), brain–blood barrier (BBB) penetration, P-glycoprotein (PGP) as an efflux transporter that can pump drugs out of cells, lipophilicity (WlogP), and polarity (TPSA) were calculated. The white region is the physicochemical space of molecules with the highest probability of being absorbed by the gastrointestinal tract that is extrapolated for dissolving semen, and the yellow region (yolk) is the physicochemical space of molecules with the highest probabilities of permeating the brain that is extrapolated for cytoplasmic penetration of spermatozoa. The yolk and white areas are compatible properties for computing the polarity and lipophilicity of small molecules. Note: ethylene glycol (EG; molecule 1); glycerol (Gly; molecule 2); soybean lecithin (Sln; molecule 3 is out of the range); fructose (Fru; molecule 4); glucose (Glc; molecule 5 is located in the same place as molecule 4); citric acid (CA; molecule 6); glutamine (Gln; molecule 7); glutathione (GSH; molecule 8 is shown without name on the right side); dimethyl formamide (DMF; molecule 9 located under molecule 12); methyl formamide (MF; molecule 10); dimethyl sulfoxide (DMSO; molecule 11); dimethylacetamide (DMA; molecule 12); methylacetamide (MA; molecule 13). GSH was out of range in the boiled-egg construction.

All penetrating CPAs show optimal ranges for the physicochemical properties, except GSH, which deviates in terms of flexibility and polarity (Figure 3). Sln is a non-penetrating CPA that deviates in terms of size, insolubility, lipophilicity, and flexibility (Figure 3). None of the CPAs except DMSO exhibit predicted high BBB penetration, as demonstrated by their location inside the yellow ellipse (yolk) outlining BBB absorption (Figure 3). Glc is located in the outer gray area, indicating that it is a compound with lower HIA and limited BBB penetration. Many CPAs are located at the boundary between the gray and white sections. Gln is situated in the white area and represents a molecule with the highest probability of passive HIA. GSH is out of range of the boiled-egg scheme. None of the CPAs are PGP^+^, and they cannot interfere with the transfer of other drugs.

### 3.3 Thermodynamics of CPAs used for SC/SV of carnivores

**FIGURE 4:**
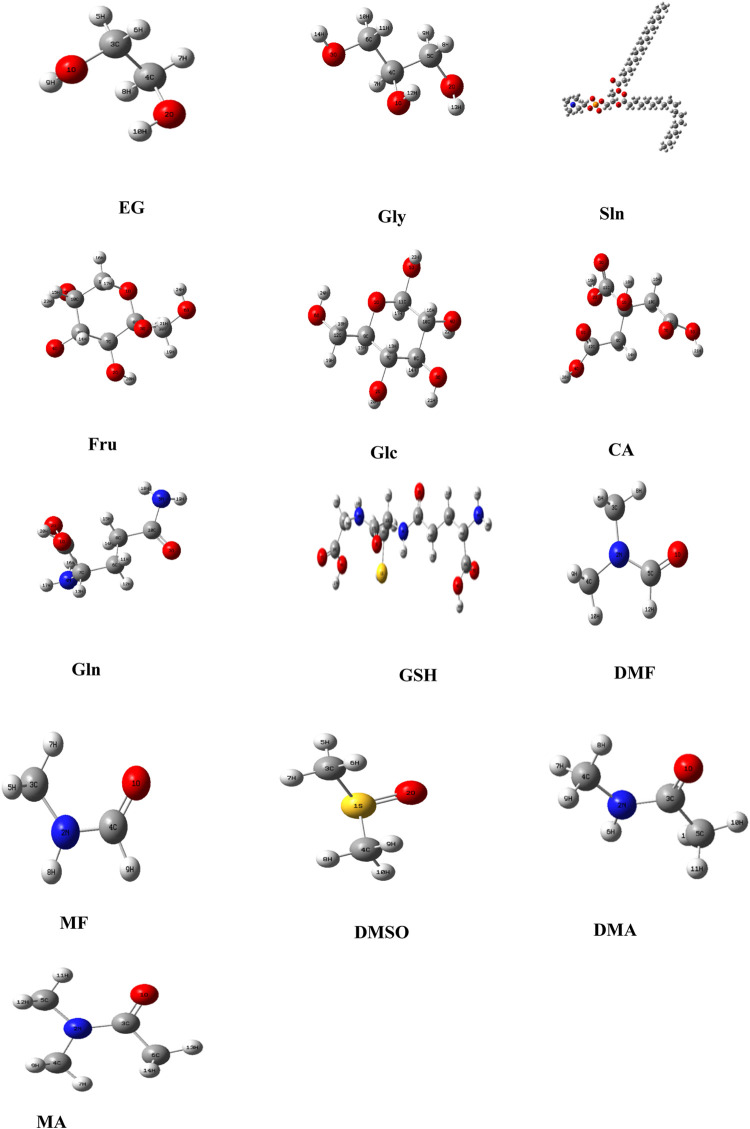
Optimized geometries of the selected cryoprotectants used in carnivorous semen cryopreservation/vitrification based on DFT/B3LYP-6311++. Note: Ethylene glycol (EG); glycerol (Gly); soybean lecithin (Sln); fructose (Fru); glucose (Glc); citric acid (CA); glutamine (Gln); glutathione (GSH); dimethyl formamide (DMF); methyl formamide (MF); dimethyl sulfoxide (DMSO); dimethylacetamide (DMA); methylacetamide (MA).

Based on the computational thermodynamic properties, Fru, Glc, and CA do not show proton affinities, whereas EG and Gly are protonated. Figure 4 predicts the optimized geometries of common small-molecule CPAs used for carnivorous SC/SV. At 813° K, the enthalpy of Gly is 3.5% higher than those of DMF and DMSO, whereas that of EG is 1.46% lower in the gaseous phase. Although most exothermic (ΔfH° < 0) cases are spontaneous, certain endothermic (ΔfH° > 0) cases can be included in this as well. Entropy is a thermodynamic parameter that describes the number of possible arrangements for a system in a particular state. The molecular structures demonstrate the increases in randomness of the particles upon melting of the solid, particularly as the liquid vaporizes (entropy S°_gas_ >> S°_liquid_ > S°_solid_). The entropy of EG S°_gas_ (311.8 J/mol k) is higher than that of S°_liquid_ (166.9 J/mol k). The Gibbs free energy (ΔfG° = ΔfH° - T S°) is calculated by combining the effects of enthalpy (ΔfH°) and entropy (S°) on a process. The ΔfG° (813 K) values for all CPAs are spontaneously formed at this temperature because the Gibbs free energies of the CPAs are less than zero. The Gibbs free energy of CA (−931.5 kJ/mol) is higher than those of all other compounds.

Table 3 shows that EG has the lowest proton affinity (PAff) among the CPAs. The PAff of Gln (937.8 kJ/mol) is greater than that of DMSO (832.1 kJ/mol); however, when the alkyl chains are lengthened, the PAff cross (at the butyl acetate and methyl pentanoate pair) becomes slightly smaller for acetates with long chains. Because the protons are accompanied by the absorption of HO⁻ groups, the Fru, Glc, and CA systems have low affinities, with the molecules collecting 20–25 times more on the inside than outside.

**TABLE 3:** Thermodynamic properties of cryoprotectants used in carnivorous semen cryopreservation based on Chemeo, high-quality chemical properties (www.chemeo.com), and Joback method predicted at 813.3 °K.

Thermodynamic property	EG	Gly	Fru	Glc	CA	Gln	DMF	MF	DMSO	DMA	MA
Proton affinity (PAff) kJ/mol	815.9	874.8	—	—	—	937.8	887.5	851.3	884.4	908.00	888.50
Enthalpy (ΔfH°) gas kJ/mol	−390.3	−576.9	−1,056.7	−1,035.02	−1,122	−461.6	−123	−116	−150.5	−170.94	−248.00 ± 5.50
Enthalpy (ΔfH°) kJ/mol liquid	−455.8	−669.6	—	—	—	—	−239	-	−203.4	[–300.10; −278.30]	−313.20 ± 1.30
Enthalpy (ΔfH°) kJ/mol solid			−1,265.6	-	−1,543	—	—	—	—	—	−318.80 ± 5.10
Molar entropy (S° gas) J/mol·K	311.8	37.8	—	—	—	—	—	—	188.78	—	—
Molar entropy (S° liquid) J/K·mol	166.9	—	—	—	—	—	—	—	—	—	—
Molar entropy (S° solid) J/K·mol	—	—	—	—	252.1	195.06	—	—	—	—	—
Gibbs free energy kJ/mol (ΔfG°)	−307.6	−438.52	−820.7	−793.7	−931.5	−272.9	−14.3	−44.1	−251.7	−35.34	−65.15

Note: ethylene glycol (EG); glycerol (Gly); soybean lecithin (Sln); fructose (Fru); glucose (Glc); citric acid (CA); glutamine (Gln); glutathione (GSH); dimethyl formamide (DMF); methyl formamide (MF); dimethyl sulfoxide (DMSO); dimethylacetamide (DMA); methylacetamide (MA).

### 3.4 ADMET and toxicity of CPAs applied for SC/SV of carnivores

Based on the data shown in Table 4, all CPAs reported for carnivorous SC/SV are healthy for the brain except GSH, DMF, MF, and DMSO, which show tendencies to be PGP (transmembrane efflux pump) substrates. None of the CPAs were PGP I and PGP II inhibitors. None of the selected CPAs could penetrate skin, and their logKp values were negative, indicating that they cannot cross skin tissue upon accidental skin exposure. Because the logKp values of all CPAs do not exceed −2.5, they do not have excellent skin permeability. The intestinal absorption percentages of CA and GSH are 0, whereas those of DMSO and Sln are 100% through the intestinal tract (Table 4). In particular, EG, DMSO, DMF, MF, Gly, and Sln show Caco2 permeabilities in this order, whereas Fru, GSH, Gln, Glc, and CA are not Caco2-permeable (Table 4). EG, DMSO, DMF, MF, Fru, and Gly are water-soluble compounds, whereas Sln, Glc, CA, Gln, and GSH show poor water solubilities (Table 4).

**TABLE 4:** Pharmacokinetic properties of transmembrane transport and penetration of selected small-molecule cryoprotectants used in carnivorous semen cryopreservation/vitrification.

Absorption	EG	Gly	Sln	Fru	Glc	CA	Gln	GSH	DMF	MF	DMSO	DMA	MA
Water solubility (log mol/L)	1.31	0.881	−3.43	0.574	−0.754	−1.423	−2.889	−2.892	0.644	0.964	0.35	0.155	0.49
Caco2 permeability (log(Papp) in 10^–6^ cm/s)	1.552	1.073	0.611	−0.616	−0.296	−0.24	−0.305	−0.536	1.479	1.462	1.497	1.488	1.472
Intestinal absorption (human) (% absorbed)	86.376	74.246	100	26.108	21.517	0	41.217	0	100	98.803	100	100	98.692
Skin permeability (logKp)	−4.031	−4.251	−2.735	−4.338	−3.253	−2.735	−2.735	−2.735	−3.058	−3.248	−2.871	−3.23	−3.417
P-glycoprotein substrate	No	No	No	No	No	No	No	Yes	Yes	Yes	Yes	Yes	Yes
P-glycoprotein I inhibitor	No	No	No	No	No	No	No	No	No	No	No	No	No
P-glycoprotein II inhibitor	No	No	Yes	No	No	No	No	No	No	No	No	No	No

Note: ethylene glycol (EG); glycerol (Gly); soybean lecithin (Sln); fructose (Fru); glucose (Glc); citric acid (CA); glutamine (Gln); glutathione (GSH); dimethyl formamide (DMF); methyl formamide (MF); dimethyl sulfoxide (DMSO); dimethylacetamide (DMA); methylacetamide (MA).

It is noted that the distributions of the CPAs in various compartments may alter the rheological properties of both SP and spermatozoa during SC/SV. The low VDss values of all CPAs except Glc indicate high water solubility or high plasma protein binding because the CPAs remain predominantly in the plasma, whereas high VDss values indicate significant concentration in the tissues, for example, due to tissue binding or high lipid solubility (Table 5). The relevancy of the computational data for BBB permeability (logBB), CNS permeability (logPs), and total clearance (log mL/min/kg) of the CPAs are obscured at this step; however, similar definitions like permeability of the cell membrane of the spermatozoa may be pursued computationally in the future (Table 5).

**TABLE 5:** Distribution of selected small-molecule cryoprotectants used in carnivorous semen cryopreservation/vitrification.

Distribution	EG	Gly	Sln	Fru	Glc	CA	Gln	GSH	DMF	MF	DMSO	DMA	MA
VDss (human; log L/kg)	−0.359	−0.492	−1.043	−0.59	0.193	−0.418	−0.5	−0.37	−0.15	−0.19	−0.191	−0.122	−0.165
Fraction unbound (human; Fu)	0.849	0.895	0.198	0.839	0.805	0.51	0.47	0.46	0.80	0.82	0.779	0.767	0.791
BBB permeability (logBB)	−0.319	−0.362	−2.156	−1.503	−0.898	−1.017	−0.5	−1.0	0.022	−0.03	−0.026	−0.008	−0.06
CNS permeability (logPs)	−2.916	−3.846	−2.927	−5.544	−3.997	−3.61	−3.6	−3.9	−2.75	−2.7	−2.484	−2.749	−2.714

Note: ethylene glycol (EG); glycerol (Gly); soybean lecithin (Sln); fructose (Fru); glucose (Glc); citric acid (CA); glutamine (Gln); glutathione (GSH); dimethyl formamide (DMF); methyl formamide (MF); dimethyl sulfoxide (DMSO); dimethylacetamide (DMA); methylacetamide (MA).

All penetrating CPAs are not substrates or inhibitors of the main CYPs, whereas Sln is a CYP3A4 substrate (Table 6). None of the CPAs are renal OCT2 substrates, and the highest total clearance was found for Sln (Table 6). Computationally, the small-molecule CPAs reported for SC/SV of carnivores can be categorized into three toxicity classes. Here, EG, Gly, Glc, and CA are low-toxicity (Class I); Gln is an intermediate-toxicity (Class II); and Sln, Fru, GSH, DMF, MF, and DMSO are high-toxicity (Class III) compounds (Table 7).

**TABLE 6:** Metabolism and excretion of selected small-molecule cryoprotectants used in carnivorous semen cryopreservation/vitrification.

Metabolism/Excretion	EG	Gly	Sln	Fru	Glc	CA	Gln	GSH	DMF	MF	DMSO	DMA	MA
CYP2D6 substrate	No	No	No	No	No	No	No	No	No	No	No	No	No
CYP3A4 substrate	No	No	Yes	No	No	No	No	No	No	No	No	No	No
CYP1A2 inhibitor	No	No	No	No	No	No	No	No	No	No	No	No	No
CYP2C19 inhibitor	No	No	No	No	No	No	No	No	No	No	No	No	No
CYP2C9 inhibitor	No	No	No	No	No	No	No	No	No	No	No	No	No
CYP2D6 inhibitor	No	No	No	No	No	No	No	No	No	No	No	No	No
CYP3A4 inhibitor	No	No	No	No	No	No	No	No	No	No	No	No	No
Renal OCT2 substrate	No	No	No	No	No	No	No	No	No	No	No	No	No
Total clearance (log mL/min/kg)	0.625	0.717	1.402	0.603	0.626	0.895	0.49	0.308	0.73	0.662	0.758	0.75	0.707

Note: ethylene glycol (EG); glycerol (Gly); soybean lecithin (Sln); fructose (Fru); glucose (Glc); citric acid (CA); glutamine (Gln); glutathione (GSH); dimethyl formamide (DMF); methyl formamide (MF); dimethyl sulfoxide (DMSO); dimethylacetamide (DMA); methylacetamide (MA); cytochrome (CYP).

**TABLE 7:** Toxicities of selected small-molecule cryoprotectants used in carnivorous semen cryopreservation.

Toxicity	EG	Gly	Sln	Fru	Glc	CA	Gln	GSH	DMF	MF	DMSO	DMA	MA
AMES toxicity	No	No	No	No	No	No	No	Yes	No	No	No	No	No
Maximum tolerated dose (human; log mg/kg/day)	1.783	2.033	0.424	2.315	No	0.749	No	1.104	1.21	1.285	1.161	1.206	1.28
hERG *I* inhibitor	No	No	No	No	2.016	No	1.23	No	No	No	No	No	No
hERG *II* inhibitor	No	No	No	No	No	No	No	No	No	No	No	No	No
Rat oral acute toxicity (LD50 mol/kg)	1.57	1.041	2.49	1.616	No	2.148	No	2.468	2.12	2.134	2.184	2.02	2.028
Rat oral chronic toxicity (LOAEL; (log mg/kg-bodyweight/day)	2.747	2.88	0.121	4.071	1.268	3.698	2.05	2.919	1.15	1.105	1.243	1.248	1.202
Hepatotoxicity	No	No	No	No	4.111	No	2.139	No	No	No	No	No	No
Skin sensitization	No	No	No	No	No	No	No	No	No	No	No	No	No
*Tetrahymena pyriformis* toxicity (log µg/L)	−2.23	−0.889	0.285	0.285	No	0.285	No	0.285	−1.07	−1.51	−0.655	−0.836	−1.258
Minnow toxicity (log mM)	3.956	3.495	−7.51	3.87	0.285	4.251	0.282	4.569	2.95	3.064	2.594	2.883	2.995
Class of toxicity	I	I	III	I	I	II	III	III	III	III	III	III	III

Note: ethylene glycol (EG); glycerol (Gly); soybean lecithin (Sln); fructose (Fru); glucose (Glc); citric acid (CA); glutamine (Gln); glutathione (GSH); dimethyl formamide (DMF); methyl formamide (MF); dimethyl sulfoxide (DMSO); dimethylacetamide (DMA); methylacetamide (MA); human ether-a-go-go-related gene (hERG); LD50: lethal dose 50; LOAEL: lowest observed adverse effect level.

The toxicity profiles of the CPAs are presented in Table 7; however, these data are not useful for daily clinical SC/SV and artificial insemination (AI). Among the CPAs discussed here, only GSH is positive in the AMES test (Table 7). Glc and Gln show hERG I inhibitory effects, whereas the remaining CPAs show no hERG I or hERG II inhibitory effects (Table 7). The rat oral acute toxicity (LD50 mol/kg) doses of EG and Gly are the lowest among the CPAs (Table 7), whereas the NOAEL of rat oral chronic toxicity of Sln is lowest among CPAs (Table 7). Glc and Gln show hepatotoxicity among the CPAs, whereas none of the CPAs show skin sensitization (Table 7). Sln, Fru, EG, CA, GSH, DMF, and MF show *T. pyriformis* toxicity values greater than −5 log µg/L and are therefore considered toxic (Table 7). Thus, Gly and DMSO are not toxic to *T. pyriformis* in the computational sense. Among all the CPAs, Sln is the only compound found to be toxic to minnows (Table 7).

## 4 Discussion

**FIGURE 5:**
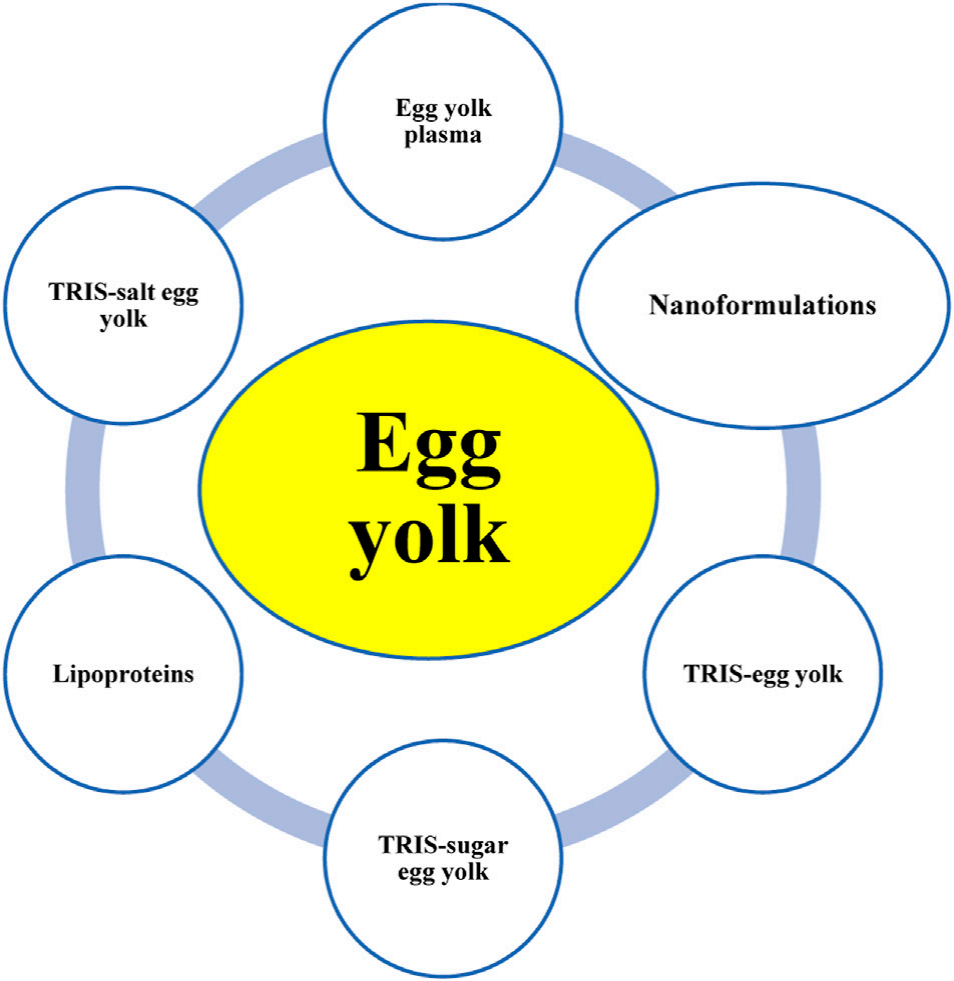
Egg-yolk-based extenders used in the semen cryopreservation/vitrification of carnivores.

Different extenders are used for carnivorous SC/SV, and various formulas are available commercially (e.g., Equex STM paste, TRIS-sugar extenders, skim milk extenders, and protein-fortified extenders); however, 42 out of 62 studies reported the egg yolk (EY)-based extenders considered in the present study. To date, EY and its byproducts, such as EY plasma (EYP), TRIS-EY, and EYs buffered with salts (e.g., citrate) have been used as dominant extenders for the SC/SV of carnivores (Figure 5). Lipid derivatives of EY, including low-density lipoprotein (LDL) and lecithin, have also been used. EY was either used as a single material for the SC/SV of carnivores or fortified with buffers, bases, salts, or sugars. To the best of our knowledge, there is no integrated study available pertaining to the rheological properties of EY or EY-based extenders and their cryoprotective potentials. EY is a viscous fluid and must be diluted before use as an extender or basic material for preparing new marketable extenders. As shown schematically in Figure 5, EY or EY-based extenders can also be mixed with other materials or extenders, including Gly, ACP-160 C^®^, sugars like Fru and Glc, bases like TRIS and citrate, and botanicals like soybean-based biomaterials, in various proportions. However, researchers have focused on the biological properties of these formulations, and there is no reliable information about their biophysical properties, such as pH, viscosity, molecular weight, and biosafety.

More investigations are needed to assess the rheological, physicochemical, and toxicological properties of extenders used for the SC/SV of pet animals. For instance, polyvinyl alcohol (PVA) has been reportedly used as a non-EY-based extender (Hamad Talha et al., 2021), and its pH value for a 4% aqueous solution ranges from 5 to 8 along with strong hydrophilic behaviors (PubChem NTP; Gudeman and Peppas, 1995). Surprisingly, PVA shows contraceptive effects when used in products designed for intravaginal administration in human women (Sanders and Matthews, 1990); therefore, its HSDB information and complete separation from semen before AI should be studied carefully. Thus, the eutectic effects of extenders and CPAs must be investigated computationally and experimentally.

Several aspects of the toxicities of CPAs were explored in a seminal paper (Best, 2015). To inhibit ice formation during cryopreservation at cryogenic temperatures, the authors focused on penetrating CPAs; this work highlights that increased CPA concentration may lead to toxicity and suggests strategies to overcome this problem by optimizing the cooling and warming rates or optimizing the time of addition of the individual CPAs during cooling. One of the most striking features reported in Best (2015) was the classification of the toxicities into specific and non-specific categories; accordingly, toxicity can be specific to a particular CPA (specific toxicity) or a consequence of being a CPA (non-specific toxicity). More specifically, CPAs are believed to prevent ice formation by interfering with the hydrogen bonding between water molecules, and this effect has been noted to cause non-specific toxicity. Herein, we discuss both specific and non-specific toxicities of CPAs to offer researchers a knowledge-based computational roadmap for designing smart extenders or CPAs. Therefore, we focus on the toxicities of common small-molecule CPAs used in carnivorous SC/SV.

It is recommended to remove CPAs during thawing and before AI; however, the success rate of this operation cannot be determined precisely. Clinicians often do not pursue this step and employ CPA-contaminated semen in AI that can cause cellular toxicities in both spermatozoa and female reproductive systems, thereby resulting in lower fertility rates or teratogenic sequelae. The latter should be discussed separately and is not a goal in our hybrid approach integrating a systematic review with computational efforts. Therefore, it is essential to compute the toxicities of CPAs or novel extenders before their use in all assisted reproductive technologies (ARTs) such as SC/SV (Dohle, 2010). To address these concerns, the physicochemical, thermodynamic, and toxicological properties of small-molecule CPAs that have contributed to the rapid evaluation, validation, and preclinical assessments of CPAs are exemplified and discussed in this work.

Generally, CPAs can be categorized into intracellular (endocellular and permeating) and extracellular (exocellular and non-permeating) types depending on their ability to penetrate the plasma membranes of spermatozoa. Both EG and Gly are permeating CPAs that can reduce the concentrations of electrolytes and prevent cell shrinkage in a hypertonic solution (Zhmakin, 2009). If intracellular CPAs cannot efflux the cell quickly enough during thawing, free water may rush into the spermatozoa and the resulting cell swelling may cause cytolysis (Zhmakin, 2009). In this context, molecular PSA, i.e., the surface area of the polar atoms, is a descriptor that correlates passive molecular transport through the membrane for predicting the transport property of a chemical (Ertl et al., 2000). The TPSA of both EG and Gly is less than 100 Å^2^; therefore, they can cross the cell membrane more easily than CPAs with TPSAs greater than 100 Å^2^. Interestingly, the wide range of TPSAs of the CPAs used for carnivores has led us to conclude that CPAs cannot be categorized based on their TPSA datasets because they do not represent a rule of thumb. The hydroxyl groups of EG or Gly make them polar substances that allow penetration of the cell membrane because of their low molecular weights. The molecular mass of a penetrating CPA is typically less than 100 Da (Wowk, 2007); however, we found that other physicochemical properties could facilitate CPA penetration of the spermatozoa. The melting point of a semen sample decreases after appropriate mixing with a CPA; however, the impact of the melting point of a CPA on spermatozoa must be discussed. The freezing point depression of a semen sample is dependent on the concentrations of the added solutes; however, its dependency on the innate melting or freezing points of the added CPAs has not been discussed experimentally. Thus, EG, MF, and DMF should be under the ice point (0°C) among the selected CPAs. The prediction of the cryogenic potential of a CPA is based on its innate or acquired physicochemical properties, which require more thermodynamic investigations.

Based on the results of the computational thermodynamic properties, Fru, Glc, and CA do not show proton affinities, whereas EG and Gly are protonated. As a result, the low-affinity systems of Fru, Glc, and CA are not more diffusional but rather dependent on metabolic energy and hence transported actively. When a system in a state of dynamic equilibrium is acted upon by stress (e.g., a change in concentration, pressure, or temperature), the equilibrium will change the temperature to minimize the effects of the stress, according to Le Châtelier’s principle (Quílez-Pardo and Solaz-Portolés, 1995). Therefore, thermodynamic studies of CPAs highlight the importance of using temperature-dependent thermal characteristics to forecast the thermal history accurately.

A small molecule (or metabolite) is a low-molecular-weight organic compound that is typically involved in a biological process as a substrate or product. Metabolomics frequently focuses on small molecules with masses in the range of 50–1,500 Da (https://www.ebi.ac.uk/). The molecular weights of all selected small-molecule CPAs in this work were less than 1,500 Da; therefore, as endogenous (e.g., Gly) or natural or synthetic exogenous (e.g., Sln and DMF) metabolites, these can be analyzed with software and metabolomics servers for further feature findings (data not presented here).

Among the CPAs that have been reported for carnivorous SC/SV, GSH, DMF, MF, and DMSO are PGP (transmembrane efflux pump) substrates and cannot penetrate the biological membranes (e.g., spermatozoa plasma membrane) (Mealey and Fidel, 2015) of cryopreserved semen in situations where repeated services are required. All reported CPAs were not PGP I and PGP II inhibitors in this review; therefore, they cannot interfere with the absorption of other medications by inhibiting the transmembrane efflux pump. None of the selected CPAs can penetrate skin, and if they remain in the thawed semen, their absorption through the skin tissues of the reproductive tracts of carnivores would not be problematic. To the best of our knowledge, there are no comparable and relevant data regarding the importance of logKp for CPAs. Clinically, accidental rupture of the rectal tissue during AI may be relevant to this finding, which could lead to intestinal absorption or possible specific toxicity. To investigate this, the Caco2 cell line is widely used as an *in vitro* model for predicting human drug absorption. The translation of the findings from computational and experimental data to spermatozoa permeation in the case of extracellular or intracellular CPAs requires further investigation. EG, DMSO, DMF, MF, DMA, MA, Fru, and Gly were investigated as water-soluble compounds, whereas Sln, Glc, CA, Gln, and GSH were investigated as poorly water-soluble compounds. Therefore, a drug-discovery-type approach must be used in the discovery and design of less-toxic CPAs (Murray and Gibson, 2022).

**FIGURE 6:**
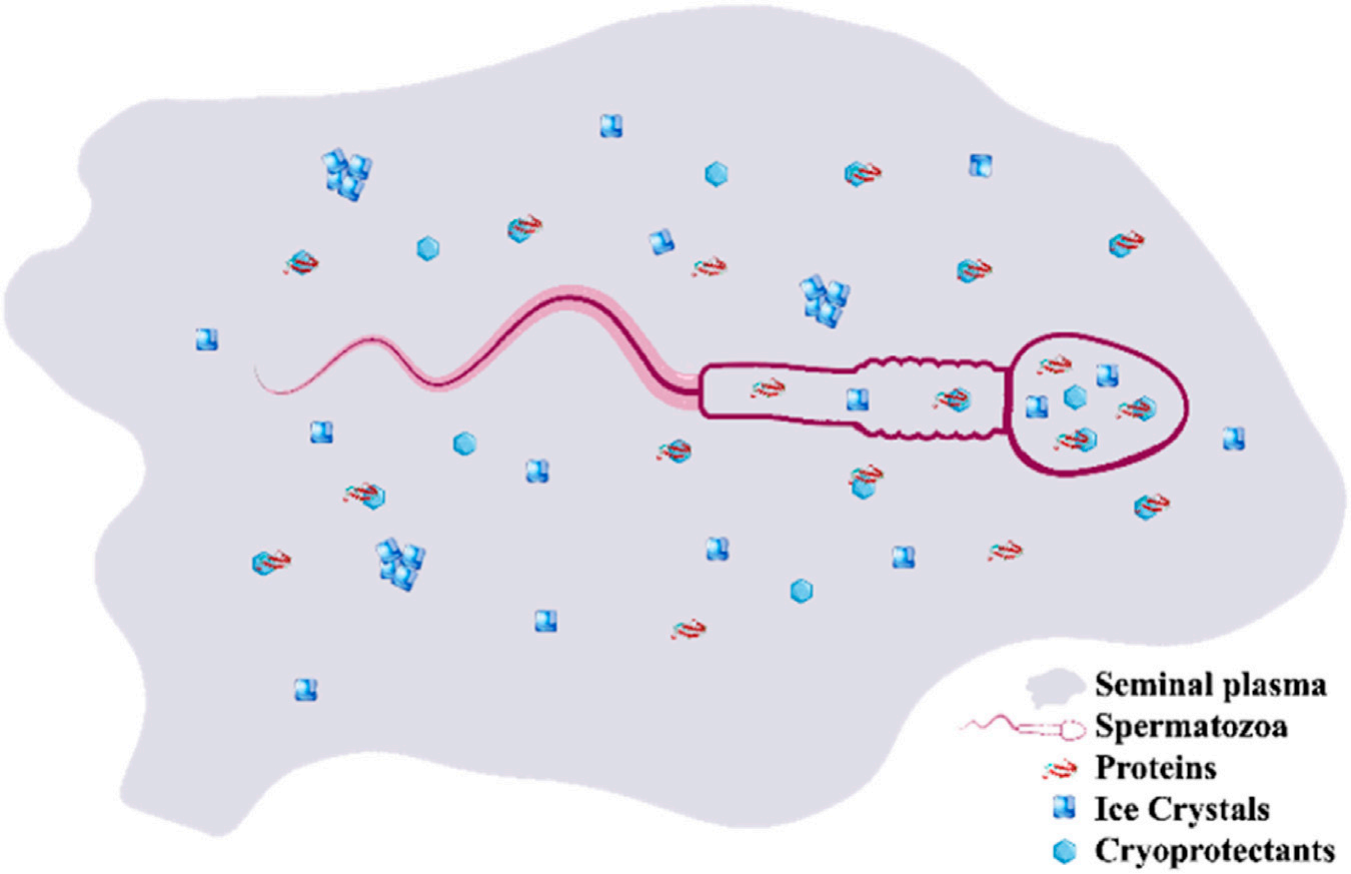
Bipartite compartment of spermatozoa and seminal plasma contains both penetrating and non-penetrating cryoprotectants that are bound or unbound to proteins of the seminal plasma or membrane of the spermatozoa.

Extrapolation of the VDss and F_u_ results to the bipartite compartment of SP and spermatozoa may be erroneous at this point; however, this comparison may provide some cues regarding the distribution of CPAs between the SP and spermatozoa. The chemical composition of SP varies among carnivores and may influence SC/SV with/without CPA addition. Primarily, SP is a heterogeneous medium containing an array of biochemical components, including ions, energy substrates, organic compounds, nitrogenous components, and reducing substances (Juyena and Stelletta, 2012; Aisen et al., 2021). We cannot rule out the interactions between the CPAs and components (mainly proteins) of the SP and spermatozoa. Therefore, the distribution of CPAs between the SP and spermatozoa cytoplasm as well as the ratios of unbounded to bounded fractions of the CPAs are unexplained aspects of SC/SV that should be modeled, computed, and validated (Figure 6). In summary, it appears that some CPAs are distributed in various compartments and could alter the rheological properties of both SP and spermatozoa during SC/SV. The low VDss values of all CPAs except Glc indicate high water solubility or high plasma protein binding because the CPAs remain predominantly in the plasma; conversely, a high VDss value implies significant concentration in the tissues, for example, due to tissue binding or high lipid solubility. The relevancy of the data obtained computationally for BBB permeability (logBB), CNS permeability (logPs), and total clearance (log mL/min/kg) for the CPAs are obscured at this step; however, similar definitions such as the blood–testis barrier, spermatozoa cell membrane permeability, and post-SC/SV alterations may be explored computationally in the future (Antonouli et al., 2024).

The cellular locations of small-molecule CPAs are predicted in this study. Accordingly, none of the CPAs are localized in the cell membrane, while EG, CA, GSH, DMF, DMA, MA, and DMSO are localized in the cytoplasm. Moreover, EG, Gly, Fru, Glc, CA, Gln, GSH, DMF, MF, and DMSO are localized in the extracellular compartment. Some CPAs are also localized in the mitochondria (e.g., Gly, CA, Gln, and GSH), lysosomes, Golgi apparatus (e.g., Glc), and endoplasmic reticulum (e.g., GSH and Glc). Therefore, the bioaccumulation of a CPA in any cellular compartment would determine its specific toxicity.

In a pioneering work, it was reported that GSH could lead to mutagenicity even at the normal levels found in mammalian tissues (Glatt et al., 1983). Glc and Gln showed hERG I inhibitory effects, whereas the remaining CPAs showed no hERG I or hERG II inhibitory effects. hERG is also expressed in the heart tissues of dogs and is known as a canonical target for screening the pro-arrhythmogenic and non-arrhythmogenic activities of HERG-blocking agents (Schneider et al., 2005). The relevancy of hERG may seem vague for the evaluation of CPA toxicity; however, when tracing the toxicodynamics of CPAs in spermatozoa, an array of ion channels such as the Na/K-ATPase (NKA) and sperm-specific cation channel (Catsper channel) (Sultana Syeda et al., 2020; Sun et al., 2017) would be candidates because their blockage interferes with the normal (electro)physiology of spermatozoa during their voyage from the testes to the fertilization sites in the uterine tubes.

## 5 Conclusion

Newtonian and non-Newtonian fluids or extenders may alter the viability of spermatozoa in different ways because they offer spermatozoa a new milieu for swimming. The extender compositions and CPAs employed in SC/SV are the main determinants of a successful ART program and can avoid iatrogenic male-factor failure in this regard. The systematic review presented herein shows that the addition of glycerol (glycerinating) and EY (luteinizing) are the two common methods of preparing semen extenders in over 60 years of research on carnivorous SC/SV. Thermoinjuries including cryoinjury and pyroinjury are the major technological and methodological barriers in the smart design of semen extenders. To overcome these thermoinjuries, the specific and non-specific toxicities of CPAs should be determined and optimized as the core concepts of cell cryopreservation. In this context, non-specific toxicity is caused by ice crystallization, while specific toxicity is dependent on the innate toxic properties of the CPAs of interest and are related to their concentrations. Herein, we attempted to include both types of toxicities of CPAs to build an applied-knowledge-based computational package for designing smart extenders or CPAs.

In the present study, we introduce several biophysical, thermodynamic, and biological features that can shed light on semen cryobiotechnology; however, the impetus to curate and integrate a reference database is heavily sensed here. When antibiotics are present in the mixture of semen and extenders, the biophysical properties of SC/SV of semen and spermatozoa may be altered in addition to the antimicrobial effects. In some cases, clinicians may have to repeat AI owing to the low infertility rates of packs or pets or the occurrences of enzootic diseases; therefore, evaluation of CPA toxicity should be considered among other factors.
